# 2-(4-{4-[4-(Oxiran-2-ylmeth­oxy)phen­oxy]phen­yl}phen­oxy­meth­yl)oxirane

**DOI:** 10.1107/S1600536812005740

**Published:** 2012-02-17

**Authors:** Tao Song, Jin-gang Liu, Shi-yong Yang

**Affiliations:** a Laboratory of Advanced Polymer Materials, Institute of Chemistry, Chinese Academy of Sciences (ICCAS), Beijing 100190, People’s Republic of China

## Abstract

In the title ep­oxy monomer, C_24_H_22_O_5_, the dihedral angle in the biphenyl residue is 3.34 (19)°, indicating a nearly coplanar conformation; this residue is not planar with the adjacent benzene ring [dihedral angle = 58.93 (14)°]. Each of the epoxide rings is disordered. Each epoxide ring was resolved over two alternative positions with site-occupancy ratios of 0.638 (10):0.362 (10) and 0.797 (9):0.203 (9).

## Related literature

For micro-electronic applications of biphenyl-type ep­oxy compounds, see: Lee & Neville (1990 ▶); Yoda (1997 ▶); Kim & Lee (2002 ▶). For related structures, see: Cho *et al.* (1999 ▶); Flippen-Anderson & Gilardi (1981 ▶).
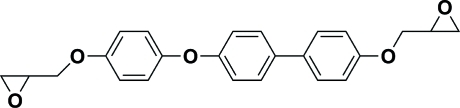


## Experimental

### 

#### Crystal data

C_24_H_22_O_5_
*M* *_r_* = 390.42
Monoclinic, 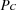

*a* = 21.261 (4) Å
*b* = 7.3103 (15) Å
*c* = 6.1322 (12) Å
β = 93.59 (3)°
*V* = 951.2 (3) Å^3^
*Z* = 2
Mo *K*α radiation
μ = 0.10 mm^−1^
*T* = 173 K
0.35 × 0.15 × 0.08 mm

#### Data collection

Rigaku Saturn724+ CCD diffractometer
Absorption correction: multi-scan (*CrystalClear*; Rigaku, 2008 ▶) *T*_min_ = 0.983, *T*_max_ = 0.992
6607 measured reflections
1965 independent reflections
1728 reflections with *I* > 2σ(*I*)
*R*_int_ = 0.065

#### Refinement

*R*[*F* ^2^ > 2σ(*F* ^2^)] = 0.085
*wR*(*F* ^2^) = 0.172
*S* = 1.18
1965 reflections
318 parameters
264 restraints
H-atom parameters constrained
Δρ_max_ = 0.23 e Å^−3^
Δρ_min_ = −0.19 e Å^−3^

### 

Data collection: *CrystalClear* (Rigaku, 2008 ▶); cell refinement: *CrystalClear*; data reduction: *CrystalClear*; program(s) used to solve structure: *SHELXS97* (Sheldrick, 2008 ▶); program(s) used to refine structure: *SHELXL97* (Sheldrick, 2008 ▶); molecular graphics: *Mercury* (Macrae *et al.*, 2006 ▶); software used to prepare material for publication: *SHELXL97*.

## Supplementary Material

Crystal structure: contains datablock(s) I, global. DOI: 10.1107/S1600536812005740/tk5057sup1.cif

Structure factors: contains datablock(s) I. DOI: 10.1107/S1600536812005740/tk5057Isup2.hkl

Supplementary material file. DOI: 10.1107/S1600536812005740/tk5057Isup3.cml

Additional supplementary materials:  crystallographic information; 3D view; checkCIF report

## Figures and Tables

**Fig. 1.**
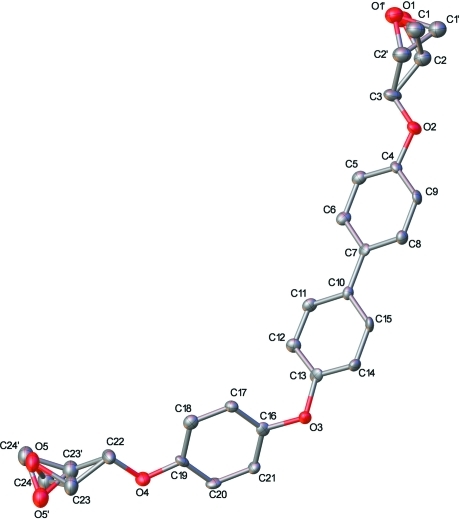
The molecular structure of (I) showing the atom numbering scheme. Displacement ellipsoids are drawn at the 30% probability level. H atoms are not shown. The atoms labelled with a prime correspond to the minor components of the disordered residues.

## supplementary crystallographic information

### Comment

The title compound can be used as matrix for epoxy coating, paintings, composites and adhesives (Lee & Neville, 1990). Especially, this compound is a very useful component for advanced epoxy molding compounds (EMCs) for microelectronic packaging. The asymmetric crystalline nature of the title epoxy compound endows the obtained EMCs with very low melting viscosity, thus providing good flow-ability during molding process (Kim & Lee, 2002). Good flow-ability of the EMCs is crucial for the packaging of thin type microelectronic devices (Yoda, 1997).

The title compound has an asymmetrical structure. The dihedral angle between the benzene ring (C16—C21) and the biphenyl ring is 58.93 (14)°. The dihedral angle in the biphenyl moiety is 3.34 (19)°, indicating a essentially co-planar conformation.

As can be seen from Fig. 1, the epoxide rings in molecule (I) are locally disordered. The two disordered residues consist of two alternative orientations for the epoxide rings connected to C3 and C22, respectively. Such disorder for epoxide rings has been noted in the literature (Cho *et al.*, 1999; Flippen-Anderson & Gilardi, 1981).

### Experimental

4-(*p*-Hydroxyphenoxy)-4'-hydroxybiphenyl (27.8 g, 0.1 mol), epichlorohydrin (222 g, 2.4 mol), and the phase transfer agent, benzyltrimethylammonium chloride (BTMAC, 0.216 g, 1 mmol) were put into a 250 ml four-necked flask equipped with a stirrer, a drop funnel, a condenser, and a thermometer. The reaction mixture was then heated to reflux for 60 min. until a homogeneous solution formed. An aqueous solution of NaOH (45 wt%, 20 g) was added drop wise over 3 h at 333 K. Then, the reaction was heated under reflux for 2 h and cooled to room temperature. The obtained white precipitate was filtered, washed with deionized water and dried *in vacuo* at 353 K overnight. The white crystalline powder was further purified by recrystallization from acetonitrile to afford the title compound (29.2 g, 75% yield). Elemental analysis: calculated for C_24_H_22_O_5_: C, 73.83; H, 5.68%. Found: C, 73.73; H, 5.69%. EI—MS, *m/z*: 390(100, *M*^+^). Colourless crystals were grown by slow evaporation of an acetone-diethyl ether solution (1:3, volume ratio) over a period of several days, M.pt:. 438 K.

### Refinement

H atoms were positioned geometrically (C—H = 0.95–1.00 Å) and refined using a riding model with the *U*_iso_(H) = 1.2*U*_eq_(C).

### Crystal data

**Table:**

C_24_H_22_O_5_	*F*(000) = 412
*M**_r_* = 390.42	*D*_x_ = 1.363 Mg m^−^^3^
Monoclinic, *P**c*	Melting point: 438 K
Hall symbol: P -2yc	Mo *K*α radiation, λ = 0.71073 Å
*a* = 21.261 (4) Å	Cell parameters from 2903 reflections
*b* = 7.3103 (15) Å	θ = 1.9–27.4°
*c* = 6.1322 (12) Å	µ = 0.10 mm^−^^1^
β = 93.59 (3)°	*T* = 173 K
*V* = 951.2 (3) Å^3^	Rod, colourless
*Z* = 2	0.35 × 0.15 × 0.08 mm

### Data collection

**Table:**

Rigaku Saturn724+ CCD diffractometer	1965 independent reflections
Radiation source: fine-focus sealed tube	1728 reflections with *I* > 2σ(*I*)
Graphite monochromator	*R*_int_ = 0.065
Detector resolution: 28.5714 pixels mm^-1^	θ_max_ = 26.5°, θ_min_ = 2.8°
ω scans at fixed χ = 45°	*h* = −26→26
Absorption correction: multi-scan (*CrystalClear*; Rigaku, 2008)	*k* = −9→8
*T*_min_ = 0.983, *T*_max_ = 0.992	*l* = −6→7
6607 measured reflections	

### Refinement

**Table:**

Refinement on *F*^2^	Primary atom site location: structure-invariant direct methods
Least-squares matrix: full	Secondary atom site location: difference Fourier map
*R*[*F*^2^ > 2σ(*F*^2^)] = 0.085	Hydrogen site location: inferred from neighbouring sites
*wR*(*F*^2^) = 0.172	H-atom parameters constrained
*S* = 1.18	*w* = 1/[σ^2^(*F*_o_^2^) + (0.0442*P*)^2^ + 0.969*P*] where *P* = (*F*_o_^2^ + 2*F*_c_^2^)/3
1965 reflections	(Δ/σ)_max_ = 0.001
318 parameters	Δρ_max_ = 0.23 e Å^−^^3^
264 restraints	Δρ_min_ = −0.19 e Å^−^^3^

### Special details

**Table:**

Geometry. All e.s.d.'s (except the e.s.d. in the dihedral angle between two l.s. planes) are estimated using the full covariance matrix. The cell e.s.d.'s are taken into account individually in the estimation of e.s.d.'s in distances, angles and torsion angles; correlations between e.s.d.'s in cell parameters are only used when they are defined by crystal symmetry. An approximate (isotropic) treatment of cell e.s.d.'s is used for estimating e.s.d.'s involving l.s. planes.
Refinement. Refinement of *F*^2^ against ALL reflections. The weighted *R*-factor *wR* and goodness of fit *S* are based on *F*^2^, conventional *R*-factors *R* are based on *F*, with *F* set to zero for negative *F*^2^. The threshold expression of *F*^2^ > σ(*F*^2^) is used only for calculating *R*-factors(gt) *etc*. and is not relevant to the choice of reflections for refinement. *R*-factors based on *F*^2^ are statistically about twice as large as those based on *F*, and *R*- factors based on ALL data will be even larger.

### Fractional atomic coordinates and isotropic or equivalent isotropic displacement parameters (Å^2^)

**Table:**

	*x*	*y*	*z*	*U*_iso_*/*U*_eq_	Occ. (<1)
O2	0.9679 (2)	0.7379 (6)	0.1402 (7)	0.0392 (11)	
O3	0.54379 (19)	0.7479 (6)	−0.6168 (6)	0.0332 (10)	
O4	0.3068 (2)	0.7607 (6)	−0.2978 (7)	0.0410 (11)	
O1	1.0723 (6)	0.8198 (13)	0.6156 (15)	0.061 (2)	0.638 (10)
C1	1.0717 (6)	0.6394 (13)	0.5111 (18)	0.060 (2)	0.638 (10)
H1B	1.1127	0.5836	0.4810	0.072*	0.638 (10)
H1A	1.0386	0.5516	0.5492	0.072*	0.638 (10)
C2	1.0523 (4)	0.7999 (16)	0.3903 (17)	0.055 (2)	0.638 (10)
H2	1.0802	0.8476	0.2777	0.066*	0.638 (10)
O1'	1.0674 (10)	0.770 (3)	0.653 (2)	0.060 (3)	0.362 (10)
C1'	1.0982 (9)	0.767 (3)	0.449 (3)	0.059 (3)	0.362 (10)
H1'B	1.1318	0.6756	0.4321	0.070*	0.362 (10)
H1'A	1.1042	0.8853	0.3743	0.070*	0.362 (10)
C2'	1.0322 (7)	0.700 (2)	0.466 (3)	0.058 (3)	0.362 (10)
H2'	1.0254	0.5642	0.4592	0.070*	0.362 (10)
C3	0.9816 (3)	0.8206 (11)	0.3504 (11)	0.0502 (19)	
H3A	0.9591	0.7566	0.4646	0.060*	
H3B	0.9692	0.9512	0.3485	0.060*	
C4	0.9072 (2)	0.7504 (8)	0.0492 (9)	0.0279 (13)	
C5	0.8585 (3)	0.8380 (8)	0.1427 (9)	0.0354 (15)	
H5	0.8656	0.8995	0.2784	0.042*	
C6	0.7988 (3)	0.8361 (8)	0.0377 (9)	0.0346 (14)	
H6	0.7654	0.8960	0.1051	0.041*	
C7	0.7856 (3)	0.7487 (8)	−0.1651 (9)	0.0267 (12)	
C8	0.8369 (3)	0.6606 (8)	−0.2527 (9)	0.0317 (14)	
H8	0.8308	0.5998	−0.3893	0.038*	
C9	0.8956 (3)	0.6597 (8)	−0.1472 (9)	0.0300 (13)	
H9	0.9289	0.5957	−0.2098	0.036*	
C10	0.7221 (3)	0.7498 (7)	−0.2772 (9)	0.0263 (12)	
C11	0.6702 (3)	0.8303 (8)	−0.1862 (9)	0.0336 (14)	
H11	0.6764	0.8877	−0.0475	0.040*	
C12	0.6101 (3)	0.8307 (8)	−0.2881 (10)	0.0343 (14)	
H12	0.5760	0.8871	−0.2209	0.041*	
C13	0.6008 (3)	0.7464 (8)	−0.4914 (9)	0.0313 (14)	
C14	0.6509 (3)	0.6685 (8)	−0.5893 (9)	0.0299 (13)	
H14	0.6449	0.6136	−0.7295	0.036*	
C15	0.7100 (3)	0.6714 (8)	−0.4809 (9)	0.0315 (13)	
H15	0.7441	0.6166	−0.5500	0.038*	
C16	0.4871 (3)	0.7515 (8)	−0.5173 (9)	0.0294 (13)	
C17	0.4759 (3)	0.6561 (8)	−0.3279 (9)	0.0292 (12)	
H17	0.5092	0.5914	−0.2517	0.035*	
C18	0.4161 (3)	0.6554 (8)	−0.2499 (10)	0.0370 (14)	
H18	0.4082	0.5906	−0.1202	0.044*	
C19	0.3672 (3)	0.7514 (8)	−0.3645 (9)	0.0311 (14)	
C20	0.3787 (3)	0.8422 (8)	−0.5512 (9)	0.0349 (14)	
H20	0.3453	0.9050	−0.6294	0.042*	
C21	0.4385 (3)	0.8444 (8)	−0.6291 (9)	0.0299 (13)	
H21	0.4460	0.9096	−0.7589	0.036*	
C22	0.2927 (3)	0.6724 (9)	−0.1012 (10)	0.0426 (16)	
H22B	0.3163	0.7297	0.0254	0.051*	
H22A	0.3041	0.5412	−0.1060	0.051*	
O5	0.2061 (4)	0.6830 (10)	0.1419 (11)	0.061 (2)	0.797 (9)
C23	0.2222 (4)	0.6948 (12)	−0.0834 (13)	0.056 (2)	0.797 (9)
H23	0.1924	0.6448	−0.2014	0.067*	0.797 (9)
C24	0.2043 (5)	0.8605 (11)	0.0384 (15)	0.063 (2)	0.797 (9)
H24B	0.2380	0.9489	0.0829	0.075*	0.797 (9)
H24A	0.1626	0.9157	0.0004	0.075*	0.797 (9)
O5'	0.1776 (12)	0.714 (4)	−0.023 (4)	0.063 (3)	0.203 (9)
C23'	0.2400 (10)	0.788 (4)	−0.012 (6)	0.059 (3)	0.203 (9)
H23A	0.2427	0.9221	−0.0413	0.071*	0.203 (9)
C24'	0.207 (2)	0.737 (8)	0.193 (4)	0.061 (3)	0.203 (9)
H24C	0.2210	0.6258	0.2739	0.073*	0.203 (9)
H24D	0.1915	0.8373	0.2843	0.073*	0.203 (9)

### Atomic displacement parameters (Å^2^)

**Table:**

	*U*^11^	*U*^22^	*U*^33^	*U*^12^	*U*^13^	*U*^23^
O2	0.028 (2)	0.047 (3)	0.042 (3)	0.011 (2)	−0.0011 (18)	−0.008 (2)
O3	0.030 (2)	0.042 (3)	0.028 (2)	−0.0026 (18)	0.0013 (17)	−0.0061 (18)
O4	0.040 (3)	0.042 (3)	0.041 (2)	0.006 (2)	0.001 (2)	0.011 (2)
O1	0.060 (4)	0.069 (5)	0.053 (4)	0.006 (4)	−0.008 (3)	−0.003 (4)
C1	0.057 (4)	0.064 (5)	0.058 (4)	0.004 (4)	−0.005 (4)	−0.005 (4)
C2	0.053 (4)	0.063 (5)	0.048 (4)	0.002 (4)	−0.001 (4)	−0.003 (4)
O1'	0.058 (4)	0.069 (5)	0.052 (5)	0.005 (4)	−0.003 (4)	−0.003 (4)
C1'	0.056 (5)	0.067 (5)	0.052 (5)	0.003 (4)	−0.001 (4)	0.000 (4)
C2'	0.056 (5)	0.064 (5)	0.053 (5)	0.003 (4)	−0.007 (4)	−0.002 (4)
C3	0.039 (4)	0.062 (5)	0.047 (4)	0.015 (3)	−0.012 (3)	−0.011 (4)
C4	0.022 (3)	0.029 (3)	0.032 (3)	0.007 (2)	0.002 (2)	0.005 (2)
C5	0.045 (4)	0.029 (3)	0.032 (3)	−0.003 (3)	0.000 (3)	0.003 (2)
C6	0.045 (4)	0.027 (3)	0.032 (3)	0.009 (3)	0.001 (3)	0.001 (3)
C7	0.031 (3)	0.024 (3)	0.025 (3)	−0.004 (2)	0.005 (2)	0.011 (2)
C8	0.033 (3)	0.032 (3)	0.031 (3)	0.004 (3)	0.007 (3)	−0.006 (2)
C9	0.024 (3)	0.033 (3)	0.035 (3)	0.009 (2)	0.013 (2)	−0.003 (3)
C10	0.031 (3)	0.022 (3)	0.026 (3)	0.003 (2)	0.008 (2)	−0.002 (2)
C11	0.044 (4)	0.026 (3)	0.030 (3)	−0.002 (3)	0.000 (3)	−0.006 (2)
C12	0.037 (4)	0.028 (3)	0.038 (3)	−0.005 (3)	−0.001 (3)	−0.005 (3)
C13	0.036 (3)	0.026 (3)	0.032 (3)	−0.018 (3)	0.004 (3)	0.002 (2)
C14	0.033 (3)	0.035 (3)	0.022 (3)	0.005 (3)	0.002 (2)	−0.006 (2)
C15	0.025 (3)	0.039 (3)	0.032 (3)	−0.003 (3)	0.016 (2)	−0.004 (3)
C16	0.036 (3)	0.020 (3)	0.033 (3)	0.001 (2)	0.003 (2)	−0.011 (2)
C17	0.028 (3)	0.028 (3)	0.031 (3)	0.000 (2)	−0.002 (2)	0.002 (2)
C18	0.045 (4)	0.030 (3)	0.036 (3)	−0.003 (3)	0.001 (3)	−0.004 (3)
C19	0.019 (3)	0.034 (3)	0.040 (3)	0.007 (2)	−0.005 (2)	−0.009 (3)
C20	0.041 (4)	0.033 (3)	0.030 (3)	0.009 (3)	−0.008 (3)	0.007 (3)
C21	0.033 (3)	0.030 (3)	0.027 (3)	0.000 (3)	0.004 (2)	0.007 (2)
C22	0.034 (3)	0.050 (4)	0.044 (4)	0.004 (3)	0.007 (3)	0.017 (3)
O5	0.057 (3)	0.071 (4)	0.058 (3)	0.011 (3)	0.024 (3)	0.009 (3)
C23	0.055 (4)	0.063 (4)	0.053 (4)	0.006 (4)	0.020 (3)	0.001 (4)
C24	0.060 (4)	0.066 (4)	0.064 (4)	0.006 (4)	0.016 (3)	0.002 (4)
O5'	0.061 (5)	0.070 (5)	0.061 (5)	0.005 (5)	0.017 (4)	0.002 (5)
C23'	0.058 (5)	0.064 (5)	0.057 (5)	0.005 (5)	0.015 (5)	0.004 (5)
C24'	0.060 (5)	0.067 (6)	0.058 (5)	0.007 (5)	0.017 (5)	0.002 (5)

### Geometric parameters (Å, º)

**Table:**

O2—C4	1.378 (7)	C11—C12	1.386 (8)
O2—C3	1.437 (7)	C11—H11	0.9500
O3—C16	1.385 (7)	C12—C13	1.393 (8)
O3—C13	1.395 (7)	C12—H12	0.9500
O4—C19	1.374 (7)	C13—C14	1.377 (8)
O4—C22	1.416 (7)	C14—C15	1.385 (8)
O1—C2	1.427 (8)	C14—H14	0.9500
O1—C1	1.466 (9)	C15—H15	0.9500
C1—C2	1.434 (13)	C16—C21	1.382 (8)
C1—H1B	0.9900	C16—C17	1.388 (8)
C1—H1A	0.9900	C17—C18	1.387 (8)
C2—C3	1.517 (8)	C17—H17	0.9500
C2—H2	1.0000	C18—C19	1.405 (8)
O1'—C2'	1.423 (10)	C18—H18	0.9500
O1'—C1'	1.444 (10)	C19—C20	1.359 (9)
C1'—C2'	1.498 (18)	C20—C21	1.386 (8)
C1'—H1'B	0.9900	C20—H20	0.9500
C1'—H1'A	0.9900	C21—H21	0.9500
C2'—C3	1.532 (10)	C22—C23	1.519 (8)
C2'—H2'	1.0000	C22—C23'	1.532 (10)
C3—H3A	0.9900	C22—H22B	0.9900
C3—H3B	0.9900	C22—H22A	0.9900
C4—C5	1.373 (8)	O5—C24	1.444 (8)
C4—C9	1.383 (8)	O5—C23	1.447 (8)
C5—C6	1.387 (8)	C23—C24	1.485 (8)
C5—H5	0.9500	C23—H23	1.0000
C6—C7	1.410 (8)	C24—H24B	0.9900
C6—H6	0.9500	C24—H24A	0.9900
C7—C8	1.404 (8)	O5'—C23'	1.430 (10)
C7—C10	1.476 (7)	O5'—C24'	1.435 (10)
C8—C9	1.369 (8)	C23'—C24'	1.526 (10)
C8—H8	0.9500	C23'—H23A	1.0000
C9—H9	0.9500	C24'—H24C	0.9900
C10—C15	1.383 (8)	C24'—H24D	0.9900
C10—C11	1.398 (8)		
			
C4—O2—C3	117.8 (4)	C13—C12—H12	120.8
C16—O3—C13	120.6 (4)	C14—C13—C12	120.2 (6)
C19—O4—C22	118.8 (5)	C14—C13—O3	115.6 (5)
C2—O1—C1	59.4 (6)	C12—C13—O3	124.0 (6)
C2—C1—O1	59.0 (5)	C13—C14—C15	119.1 (5)
C2—C1—H1B	117.9	C13—C14—H14	120.4
O1—C1—H1B	117.9	C15—C14—H14	120.4
C2—C1—H1A	117.9	C10—C15—C14	123.5 (5)
O1—C1—H1A	117.9	C10—C15—H15	118.3
H1B—C1—H1A	115.0	C14—C15—H15	118.3
O1—C2—C1	61.6 (5)	C21—C16—O3	115.8 (5)
O1—C2—C3	112.2 (9)	C21—C16—C17	120.0 (5)
C1—C2—C3	114.5 (10)	O3—C16—C17	123.9 (5)
O1—C2—H2	118.6	C18—C17—C16	119.9 (5)
C1—C2—H2	118.6	C18—C17—H17	120.0
C3—C2—H2	118.6	C16—C17—H17	120.0
C2'—O1'—C1'	63.0 (9)	C17—C18—C19	119.4 (6)
O1'—C1'—C2'	57.8 (6)	C17—C18—H18	120.3
O1'—C1'—H1'B	118.0	C19—C18—H18	120.3
C2'—C1'—H1'B	118.0	C20—C19—O4	116.6 (5)
O1'—C1'—H1'A	118.0	C20—C19—C18	119.9 (6)
C2'—C1'—H1'A	118.0	O4—C19—C18	123.5 (5)
H1'B—C1'—H1'A	115.2	C19—C20—C21	120.9 (5)
O1'—C2'—C1'	59.2 (6)	C19—C20—H20	119.6
O1'—C2'—C3	118.9 (16)	C21—C20—H20	119.6
C1'—C2'—C3	114.3 (16)	C16—C21—C20	119.8 (5)
O1'—C2'—H2'	117.2	C16—C21—H21	120.1
C1'—C2'—H2'	117.2	C20—C21—H21	120.1
C3—C2'—H2'	117.2	O4—C22—C23	105.8 (5)
O2—C3—C2	104.2 (6)	O4—C22—C23'	104.5 (14)
O2—C3—C2'	105.6 (8)	O4—C22—H22B	110.6
O2—C3—H3A	110.9	C23—C22—H22B	110.6
C2—C3—H3A	110.9	C23'—C22—H22B	80.3
C2'—C3—H3A	75.9	O4—C22—H22A	110.6
O2—C3—H3B	110.9	C23—C22—H22A	110.6
C2—C3—H3B	110.9	C23'—C22—H22A	136.9
C2'—C3—H3B	137.7	H22B—C22—H22A	108.7
H3A—C3—H3B	108.9	C24—O5—C23	61.8 (4)
C5—C4—O2	125.1 (5)	O5—C23—C24	59.0 (4)
C5—C4—C9	119.3 (5)	O5—C23—C22	110.8 (7)
O2—C4—C9	115.6 (5)	C24—C23—C22	114.1 (8)
C4—C5—C6	119.6 (5)	O5—C23—H23	119.5
C4—C5—H5	120.2	C24—C23—H23	119.5
C6—C5—H5	120.2	C22—C23—H23	119.5
C5—C6—C7	122.7 (6)	O5—C24—C23	59.2 (4)
C5—C6—H6	118.7	O5—C24—H24B	117.9
C7—C6—H6	118.7	C23—C24—H24B	117.9
C8—C7—C6	115.4 (6)	O5—C24—H24A	117.9
C8—C7—C10	122.3 (5)	C23—C24—H24A	117.9
C6—C7—C10	122.3 (5)	H24B—C24—H24A	115.0
C9—C8—C7	121.9 (5)	C23'—O5'—C24'	64.3 (6)
C9—C8—H8	119.0	O5'—C23'—C24'	58.0 (6)
C7—C8—H8	119.0	O5'—C23'—C22	118 (2)
C8—C9—C4	121.1 (5)	C24'—C23'—C22	123 (3)
C8—C9—H9	119.4	O5'—C23'—H23A	115.1
C4—C9—H9	119.4	C24'—C23'—H23A	115.1
C15—C10—C11	115.3 (5)	C22—C23'—H23A	115.1
C15—C10—C7	122.1 (5)	O5'—C24'—C23'	57.7 (5)
C11—C10—C7	122.6 (5)	O5'—C24'—H24C	118.0
C12—C11—C10	123.3 (5)	C23'—C24'—H24C	118.0
C12—C11—H11	118.3	O5'—C24'—H24D	118.0
C10—C11—H11	118.3	C23'—C24'—H24D	118.0
C11—C12—C13	118.5 (6)	H24C—C24'—H24D	115.2
C11—C12—H12	120.8		
			
C1—O1—C2—C3	106.7 (11)	C16—O3—C13—C14	154.0 (5)
O1—C1—C2—C3	−103.1 (10)	C16—O3—C13—C12	−31.6 (8)
C1'—O1'—C2'—C3	−103 (2)	C12—C13—C14—C15	1.5 (9)
O1'—C1'—C2'—C3	110.3 (19)	O3—C13—C14—C15	176.2 (5)
C4—O2—C3—C2	−174.2 (6)	C11—C10—C15—C14	−0.9 (8)
C4—O2—C3—C2'	146.9 (9)	C7—C10—C15—C14	179.3 (5)
O1—C2—C3—O2	−161.3 (8)	C13—C14—C15—C10	−0.3 (9)
C1—C2—C3—O2	−93.5 (9)	C13—O3—C16—C21	147.4 (5)
O1—C2—C3—C2'	−64.0 (15)	C13—O3—C16—C17	−38.3 (8)
C1—C2—C3—C2'	3.8 (13)	C21—C16—C17—C18	−0.5 (8)
O1'—C2'—C3—O2	163.7 (14)	O3—C16—C17—C18	−174.6 (5)
C1'—C2'—C3—O2	96.8 (14)	C16—C17—C18—C19	0.1 (8)
O1'—C2'—C3—C2	70.6 (16)	C22—O4—C19—C20	−178.4 (6)
C1'—C2'—C3—C2	3.7 (11)	C22—O4—C19—C18	1.3 (9)
C3—O2—C4—C5	0.8 (9)	C17—C18—C19—C20	0.7 (9)
C3—O2—C4—C9	−176.4 (6)	C17—C18—C19—O4	−178.9 (5)
O2—C4—C5—C6	−177.9 (5)	O4—C19—C20—C21	178.5 (5)
C9—C4—C5—C6	−0.8 (9)	C18—C19—C20—C21	−1.2 (9)
C4—C5—C6—C7	−0.7 (9)	O3—C16—C21—C20	174.6 (5)
C5—C6—C7—C8	1.0 (8)	C17—C16—C21—C20	0.1 (8)
C5—C6—C7—C10	−179.1 (5)	C19—C20—C21—C16	0.8 (9)
C6—C7—C8—C9	0.2 (8)	C19—O4—C22—C23	−175.6 (5)
C10—C7—C8—C9	−179.7 (5)	C19—O4—C22—C23'	149.6 (13)
C7—C8—C9—C4	−1.7 (9)	C24—O5—C23—C22	106.3 (8)
C5—C4—C9—C8	2.0 (9)	O4—C22—C23—O5	−155.6 (6)
O2—C4—C9—C8	179.4 (5)	C23'—C22—C23—O5	−63 (3)
C8—C7—C10—C15	−3.2 (8)	O4—C22—C23—C24	−91.2 (7)
C6—C7—C10—C15	176.9 (6)	C23'—C22—C23—C24	1 (2)
C8—C7—C10—C11	177.1 (6)	C22—C23—C24—O5	−100.7 (8)
C6—C7—C10—C11	−2.8 (8)	C24'—O5'—C23'—C22	113 (4)
C15—C10—C11—C12	1.0 (8)	O4—C22—C23'—O5'	107 (2)
C7—C10—C11—C12	−179.2 (5)	C23—C22—C23'—O5'	10.7 (14)
C10—C11—C12—C13	0.1 (9)	O4—C22—C23'—C24'	176 (2)
C11—C12—C13—C14	−1.4 (9)	C23—C22—C23'—C24'	79 (2)
C11—C12—C13—O3	−175.6 (5)	C22—C23'—C24'—O5'	−105 (3)
